# pIgR and PECAM-1 bind to pneumococcal adhesins RrgA and PspC mediating bacterial brain invasion

**DOI:** 10.1084/jem.20161668

**Published:** 2017-06-05

**Authors:** Federico Iovino, Joo-Yeon Engelen-Lee, Matthijs Brouwer, Diederik van de Beek, Arie van der Ende, Merche Valls Seron, Peter Mellroth, Sandra Muschiol, Jan Bergstrand, Jerker Widengren, Birgitta Henriques-Normark

**Affiliations:** 1 Department of Microbiology, Tumor and Cell Biology, Karolinska Institutet, SE-171 77 Stockholm, Sweden; 2 Department of Clinical Microbiology, Karolinska University Hospital, SE-171 76 Stockholm, Sweden; 3 Department of Neurology, Center for Infection and Immunity Amsterdam, Academic Medical Center, University of Amsterdam, 1012 WX Amsterdam, Netherlands; 4 Lee Kong Chian School of Medicine (LKC), Nanyang Technological University, Singapore 639798, Singapore; 5 Singapore Centre on Environmental Life Sciences Engineering (SCELSE), Nanyang Technological University, Singapore 639798, Singapore; 6 Department of Applied Physics, KTH Royal Institute of Technology, SE-114 28 Stockholm, Sweden

## Abstract

*Streptococcus pneumoniae* is the main cause of bacterial meningitis, a life-threating disease with a high case fatality rate despite treatment with antibiotics. Pneumococci cause meningitis by invading the blood and penetrating the blood–brain barrier (BBB). Using stimulated emission depletion (STED) super-resolution microscopy of brain biopsies from patients who died of pneumococcal meningitis, we observe that pneumococci colocalize with the two BBB endothelial receptors: polymeric immunoglobulin receptor (pIgR) and platelet endothelial cell adhesion molecule (PECAM-1). We show that the major adhesin of the pneumococcal pilus-1, RrgA, binds both receptors, whereas the choline binding protein PspC binds, but to a lower extent, only pIgR. Using a bacteremia-derived meningitis model and mutant mice, as well as antibodies against the two receptors, we prevent pneumococcal entry into the brain and meningitis development. By adding antibodies to antibiotic (ceftriaxone)-treated mice, we further reduce the bacterial burden in the brain. Our data suggest that inhibition of pIgR and PECAM-1 has the potential to prevent pneumococcal meningitis.

## Abstract (precis)

Pneumococci are major causes of bacterial meningitis. Iovino et al. show that pneumococci invade the brain and pass the blood–brain barrier by interacting with the endothelial receptors pIgR and PECAM-1 recognizing the pneumococcal adhesin RrgA and PspC on the bacterial surface.

## Introduction

*Streptococcus pneumoniae* is a main cause of bacterial meningitis globally, with an estimate of 100,000 cases among children younger than 5 yr (O’Brien et al., 2009). Despite access to antibiotics, mortality ranges from ∼8% to 37% depending on geographical region. Neurological sequelae such as hearing loss, focal deficits, and motor and cognitive impairments significantly affect the quality of life of survivors (van de Beek et al., 2004, 2006; O’Brien et al., 2009; Brouwer et al., 2010; Bijlsma et al., 2016). Antibiotic resistance is emerging and often caused by the spread of pneumococcal clones frequently expressing adhesive pili, promoting nasopharyngeal carriage (Barocchi et al., 2006; Sjöström et al., 2007; Moschioni et al., 2008).

Meningitis is usually caused by bacteria reaching the brain through the bloodstream. This multistep process involves mucosal colonization, invasion into the bloodstream, survival and replication of bacteria within the latter, and traversal of the blood–brain barrier (BBB). Sustained bacteremia and a threshold level of bacteremia favor bacterial penetration of the BBB, which separates the brain from circulating blood and has critical functions in protection and nutrient supply of the brain (de Vries et al., 1997; Abbott et al., 2010). Endothelial cells form the layer that lines the interior surface of the blood vessels. To invade the brain from the blood, bacteria first encounter the BBB endothelium and develop strategies to pass this barrier. Receptor-mediated transcytosis has been proposed as a mechanism used by pneumococci to cross the BBB (Cundell et al., 1995; Ring et al., 1998). The polymeric Ig receptor (pIgR) mediates transport of Igs across mucosal epithelia (Asano and Komiyama, 2011) and is involved in pneumococcal adhesion to the human nasopharyngeal epithelium (Zhang et al., 2000; Lu et al., 2003). Pneumococci adhere to and colonize the nasopharyngeal epithelium through binding of the choline-binding protein CbpA, also known as PspC, to pIgR (Zhang et al., 2000). pIgR has been shown to be expressed by brain endothelial cells and pneumococci have been found to adhere to pIgR in the BBB endothelium (Iovino et al., 2014b). Platelet endothelial cell adhesion molecule (PECAM-1) is one of the major adhesion molecules expressed by endothelial cells (Newman and Newman, 2003; Privratsky and Newman, 2014; Chistiakov et al., 2016). Recently, it was described that PECAM-1, besides its physiological functions in endothelial integrity and endothelial–leukocyte interactions (Newman and Newman, 2003; Privratsky and Newman, 2014), is expressed by brain endothelial cells and mediates adhesion of *S. pneumoniae* to the BBB endothelium (Iovino et al., 2014b).

## Results and discussion

**Figure 1.**
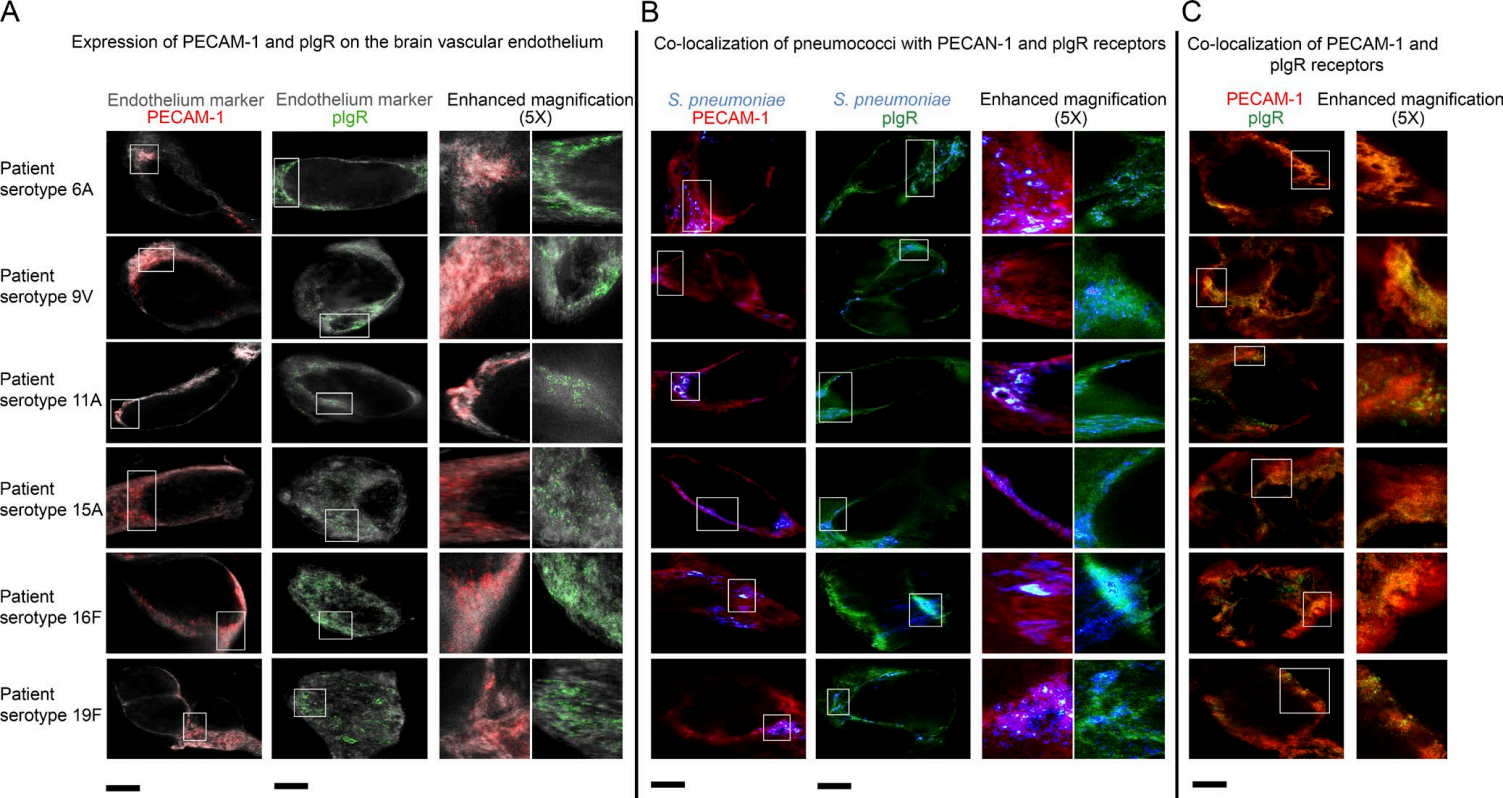
**STED super-resolution microscopy of biopsies from meningitis patients shows that pIgR and PECAM-1 are expressed on the BBB endothelium and colocalize with pneumococci.** Immunofluorescent staining using STED super-resolution microscopy of six human biopsies from patients who died of pneumococcal meningitis caused by different serotypes. Each row shows images related to the section of one blood vessel selected as representative among 10 blood vessels/patient imaged (per each staining). (A) Immunofluorescent detection of pIgR, PECAM-1, and endothelial marker shows colocalization of the two receptors on the BBB endothelium. (B) Immunofluorescent detection of *S. pneumoniae*, pIgR, and PECAM-1 shows that most pneumococci colocalize with pIgR and PECAM-1. (C) Immunofluorescent detection of pIgR and PECAM-1 shows that the two receptors colocalize in many areas of the brain vasculature. Bars, 10 µm.

Because pIgR and PECAM-1 have been suggested to act as receptors for pneumococcal entry into the brain (Iovino et al., 2014a,b), we first performed colocalization studies ex vivo using human brain autopsies. Using stimulated emission depletion (STED) super-resolution microscopy (Fig. 1) and high-resolution microscopy with the Delta Vision Elite Imaging System (Fig. S1), we performed immunofluorescent stainings of brain tissue sections from six patients who died of pneumococcal meningitis. We found that pIgR and PECAM-1 were expressed on the vascular BBB endothelium and in many areas of the brain vasculature the two receptors colocalized strongly (Fig. 1, A–C; and Fig. S1 A). Furthermore, most pneumococci that were lining the vascular BBB endothelium colocalized with pIgR and PECAM-1 (Fig. 1 B and Fig. S1 A). Quantification analysis revealed that 90–95% of all pneumococci detected colocalized with pIgR and/or PECAM-1 for all six brain tissues (Fig. S1 A) As a negative control, pneumococci did not colocalize with endothelial protein C receptor (EPCR; Fig. S1 B). Notably, superresolution STED imaging of the human brain biopsy specimens showed no signs of endothelium disruption; in fact, the fluorescent signal of the endothelium marker in gray was continuous along the endothelium layer, indicating no major disruptions (Fig. 1 A). In addition, we analyzed the integrity of the endothelial tight junctions in the blood vessels of the meningitis patients using vascular endothelial cadherin (VE-cadherin) as marker for endothelial integrity (Vestweber, 2008). Using the high-resolution Delta Vision Elite Imaging System, we performed 3D reconstruction of the blood vessels, and the images were displayed in xyz axes to give a 3D-effect visualization. We did not find that the vascular endothelium of the blood vessels was disrupted where pneumococci were attached (Fig. S1 C).

**Figure 2.**
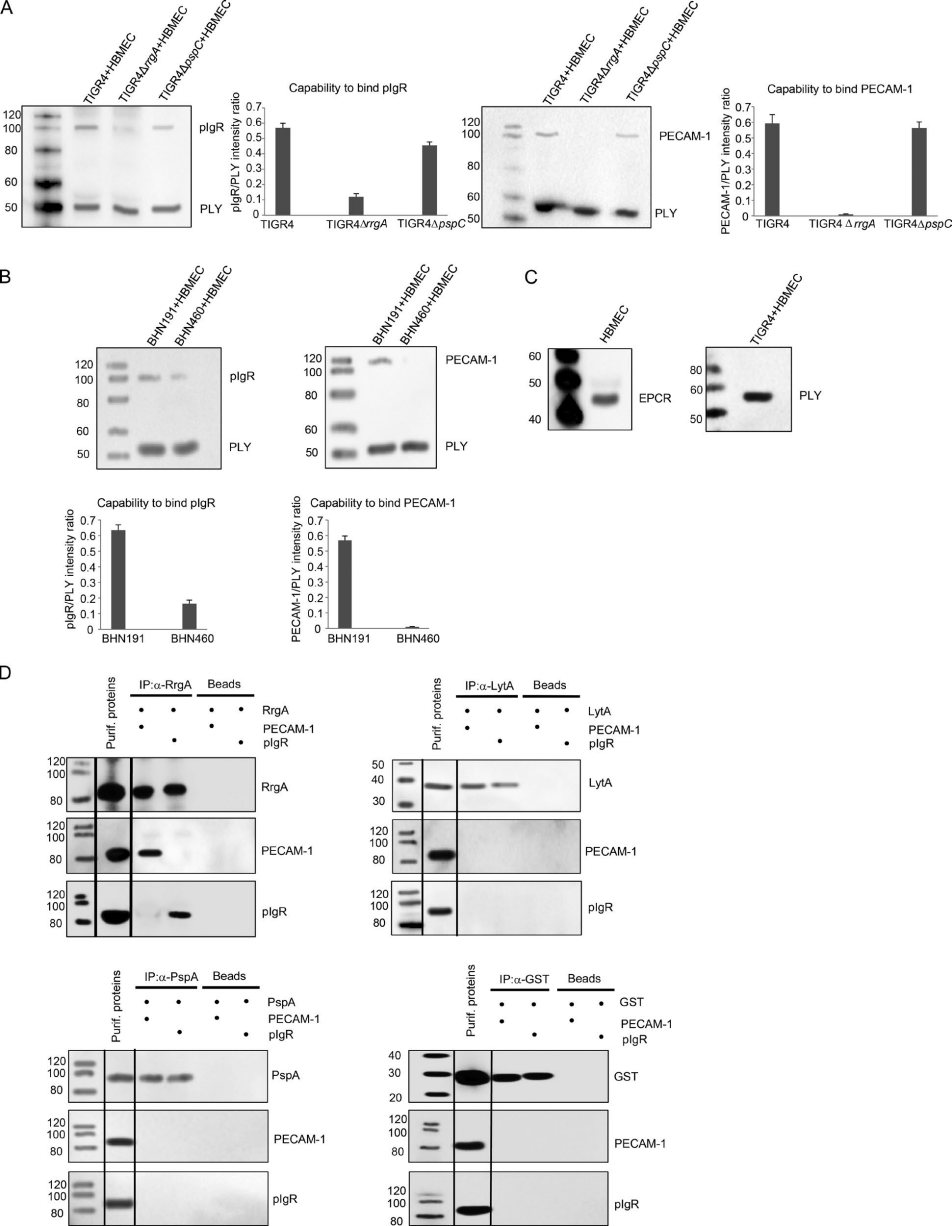
**RrgA and PspC are the pneumococcal ligands for pIgR, whereas only RrgA binds to PECAM-1 expressed by brain endothelial cells.** Incubation of TIGR4, TIGR4*ΔrrgA*, TIGR4*ΔpspC*, and the clinical isolates of serotypes 6B, piliated BHN191, and nonpiliated BHN460 with HBMEC lysate. Interactions with pIgR and PECAM-1 were detected by Western blot. (A) When TIGRΔ*pspC* is incubated with HBMEC lysate, a band of pIgR and a PECAM-1 band can be detected, indicating that without PspC, binding to pIgR and PECAM-1 still occurs. When TIGRΔ*rrgA* is incubated with HBMEC lysate, a faint band of pIgR can be detected, indicating that without RrgA, binding to pIgR is severely impaired but still occurs, whereas a PECAM-1 band is completely absent, indicating that RrgA is the major binder of PECAM-1. The pneumococcal toxin pneumolysin (PLY) was used as loading control. Quantification graphs show levels of pIgR and PECAM-1 detected on the TIGR4 strains after incubation with HBMEC lysate. (B) The same analysis was done using the clinical isolates piliated BHN191 and nonpiliated BHN460. Quantification graphs show the levels of pIgR and PECAM-1 detected on strains BHN191 and BHN460 after incubation with HBMEC lysate. The error bars show standard deviations, and each experiment has been repeated in triplicate (three biological replicates). Standard deviations have been calculated using the three measurements in each experiment. (C) The receptor EPCR is expressed by HBMECs. The pneumococcal strain TIGR4 was incubated with HBMEC lysate. No band was detected for EPCR, showing that pneumococci do not interact with EPCR. Only the loading control band for PLY was detected. Three biological replicates of each Western blot were performed, and one representative image per blot is shown. (D) Coimmunoprecipitation experiments using purified RrgA and the receptors pIgR and PECAM-1. Purified RrgA was coupled to a complex including beads and antibody, and binding of RrgA to the complex was verified by the detection of RrgA (third and fourth lanes, RrgA detection). When RrgA + complex was incubated with PECAM-1, a PECAM-1 band could be detected (third lane, PECAM-1 detection), when RrgA + complex was incubated with pIgR, a pIgR band could be detected (fourth lane, pIgR detection). As negative controls, the pneumococcal proteins PspA, LytA, and GST did not show any physical interaction with either pIgR or PECAM-1. Three biological replicates of each immunoprecipitation experiment were performed, and one representative immunoprecipitation per replicate series is shown. Black lines indicate intervening lanes have been spliced out. Molecular mass is indicated in kilodaltons.

Next, we sought to identify ligands responsible for pneumococcal–receptor interactions. Because we previously showed that the pilin adhesin protein RrgA promotes bacterial entry through the BBB (Nelson et al., 2007; Iovino et al., 2016), we first examined whether RrgA could interact with pIgR and/or PECAM-1. Using Western blots, we found that after incubation with lysates of human brain microvascular endothelial cells (HBMECs), pneumococcal strain TIGR4, expressing RrgA, bound pIgR expressed by HBMECs (Fig. 2 A). In the absence of RrgA (TIGR4Δ*rrgA*), pneumococcal binding to pIgR still occurred, but it was severely reduced (Fig. 2 A). Because pneumococcal PspC (CbpA) was previously shown to bind pIgR on nasopharyngeal epithelial cells (Zhang et al., 2000), we then incubated an isogenic mutant of TIGR4 lacking PspC (TIGR4Δ*pspC*) with HBMEC lysate. Binding to pIgR was only slightly reduced for the PspC-knockout mutant as compared with WT TIGR4 (Fig. 2 A), indicating that RrgA is the major contributor to pneumococcal binding to pIgR on the BBB. Then we studied binding to PECAM-1 and found that TIGR4 also bound this receptor after incubation with HBMEC lysates (Fig. 2 A). Deletion of RrgA (TIGR4Δ*rrgA*) completely abolished binding to PECAM-1 (Fig. 2 A), suggesting that RrgA is the major pneumococcal ligand for PECAM-1. The PspC mutant behaved similarly to WT TIGR4. In addition, we investigated the capacity of two clinical isolates of serotype 6B, the piliated meningitis isolate BHN191 and the nonpiliated isolate from a healthy carrier, BHN460 (Iovino et al., 2016), to bind to pIgR and PECAM-1 expressed by HBMECs. Piliated BHN191 bound to pIgR, whereas nonpiliated BHN460 showed a reduced and only slight binding to pIgR (Fig. 2 B). BHN191 also bound to PECAM-1 expressed by HBMECs, whereas this was not the case for nonpiliated BHN460 (Fig. 2 B). As a negative control, we found that TIGR4 did not bind to EPCR, a receptor that should not interact with pneumococci (Fig. 2 C). Collectively, these data show that on the BBB endothelium, piliated pneumococci use mainly RrgA to bind to pIgR and PECAM-1, whereas nonpiliated pneumococci use PspC to bind pIgR.

To prove direct interactions between RrgA and the receptors, we purified RrgA and performed coimmunoprecipitation experiments. We found a direct interaction between RrgA and PECAM-1, as well as with pIgR (Fig. 2 D). As a control, we used purified pneumococcal surface protein A (PspA; Ren et al., 2004), the major autolysin of *S. pneumoniae* LytA (Mellroth et al., 2014), and pneumococcal protein glutathione S-transferase (GST). No binding to PECAM-1 or pIgR was observed using these proteins (Fig. 2 D), confirming the specificity of the binding of pilin protein RrgA to PECAM-1 and pIgR.

**Figure 3.**
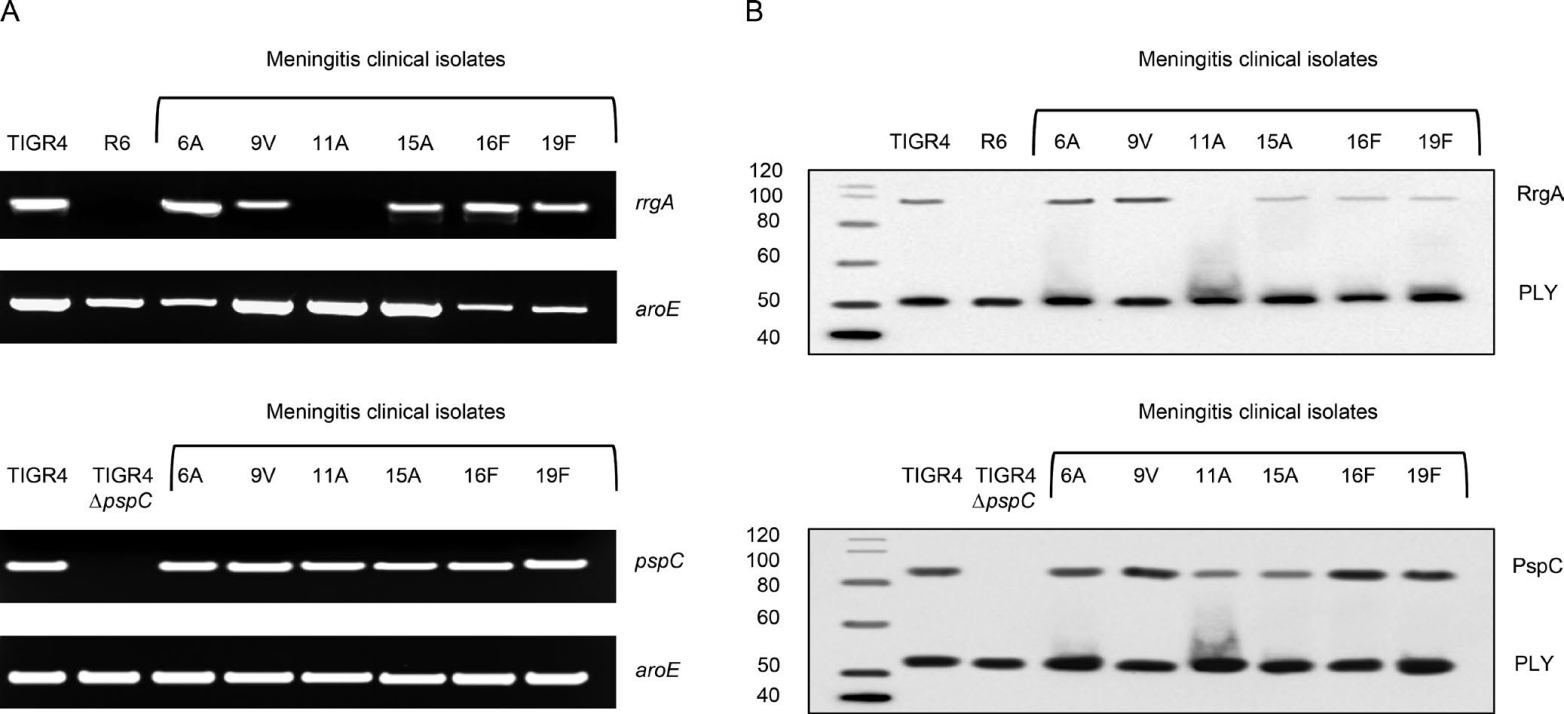
**Meningitis isolates that express RrgA and PspC bind to pIgR and PECAM-1 expressed by brain endothelial cells.** (A) PCR analysis targeting *rrgA* and *pspC* genes in the clinical isolates from six meningitis patients. *aroE* was used as a housekeeping gene control. (B) Western blot analysis to study the expression of RrgA and PspC in the six meningitis isolates. Pneumolysin (PLY) was used as loading control. For both PCR and Western blot, three biological replicates were performed, and one representative PCR gel and Western blot are shown. Molecular mass is indicated in kilodaltons.

**Figure 4.**
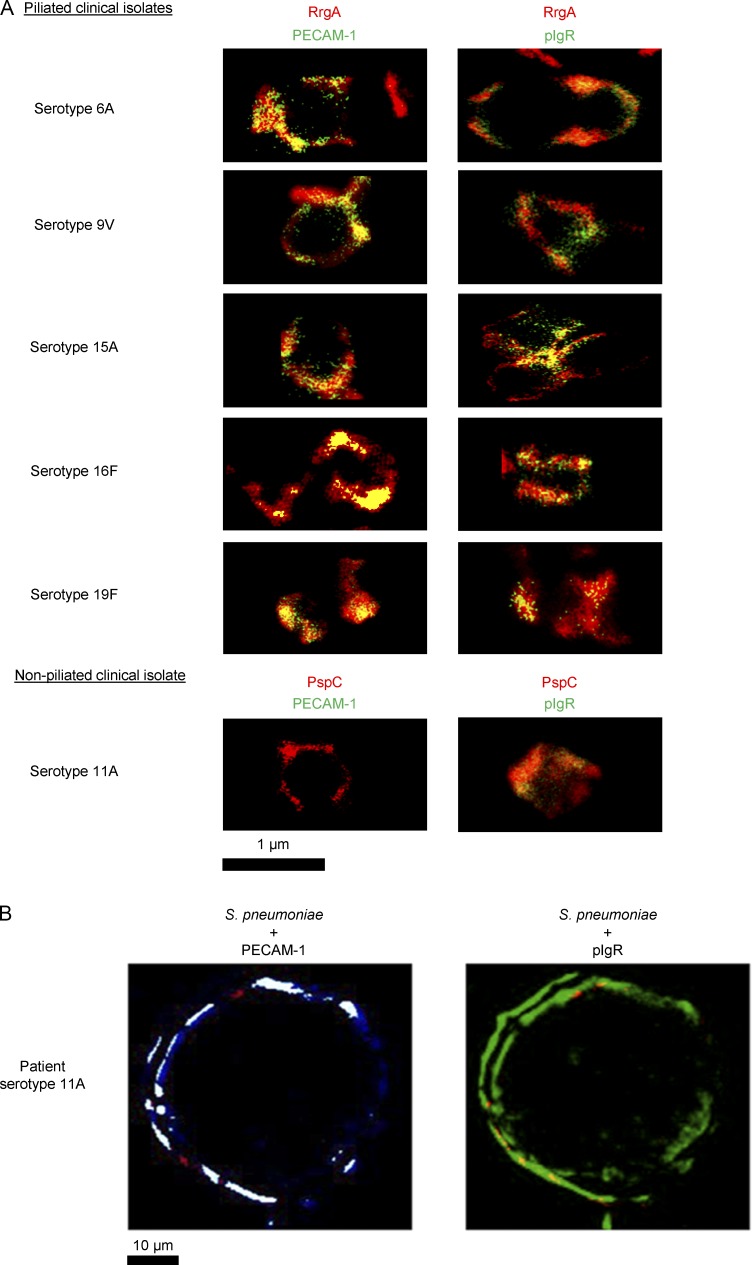
**STED super-resolution microscopy showing that endothelial pIgR and PECAM-1 colocalize with RrgA in piliated strains, whereas pIgR colocalizes with PspC in nonpiliated 11A pneumococci.** (A) STED imaging showing that pIgR and PECAM-1 from HBMEC lysate colocalize with RrgA and PspC on the five piliated clinical isolates, and only pIgR colocalizes with PspC on the nonpiliated 11A clinical isolate. For each type of staining (RrgA/PECAM-1, RrgA/pIgR, PspC/PECAM-1, and PspC/pIgR), 10 images per clinical isolate were taken, and one representative image per isolate is shown. (B) High-resolution immunofluorescent detection of *S. pneumoniae* (red), PECAM-1 (blue), and pIgR (green) in brain biopsies from a patient infected with the 11A clinical isolate. In the left panel, the portions of blood vessel in which PECAM-1 colocalizes with pIgR are shown in white. Pneumococci do not colocalize with PECAM-1 because they lack RrgA, but they colocalize with pIgR because they express PspC (right). Images are related to the section of one blood vessel selected as a representative among the 50 blood vessels of this patient that were imaged.

When we studied the presence of pili in the pneumococcal isolates recovered from the six patients that died of meningitis (see above and Fig. 1) using PCR and Western blotting, we found that five out of six isolates expressed RrgA (Fig. 3, A and B). STED microscopy showed that after incubation with HBMEC lysate, there was a colocalization between pIgR and PECAM-1 and RrgA for all five piliated clinical isolates (Fig. 4 A). Interestingly, high-resolution imaging of brain biopsies showed that the serotype 11A strain that did not express RrgA colocalized with pIgR, but not with PECAM-1, in the brain tissue (Fig. 4 B). In addition, all strains harbored PspC (Fig. 3, A and B), explaining how the nonpiliated 11A clinical isolate could invade the brain by binding to pIgR through PspC (Fig. 4). The patient infected with the nonpiliated serotype 11A strain was 88 yr old, previously severely ill, and diagnosed with inoperable cholangiocarcinoma, suggesting that underlying disease might also promote brain invasion of nonpiliated strains. This remains to be further evaluated.

Pneumococcal pili are present in ∼30% of clinical pneumococcal isolates (Barocchi et al., 2006; Nelson et al., 2007; Moschioni et al., 2008; Iovino et al., 2016), and certain serotypes and clonal types have been associated with piliation. However, we have observed that the presence and expression of pili may vary between strains, even between those belonging to the same serotype or clone (Iovino et al., 2016). This could be one explanation for the finding by Wagenvoort et al. (2016), who observed that serotypes 4 and 9V, which often are piliated, were associated with a low incidence of meningitis. Also, because all pneumococcal isolates express PspC, nonpiliated isolates may cause meningitis through binding of PspC to pIgR. Larger epidemiological studies exploring the association of pili to pneumococcal meningitis remain to be done.

**Figure 5.**
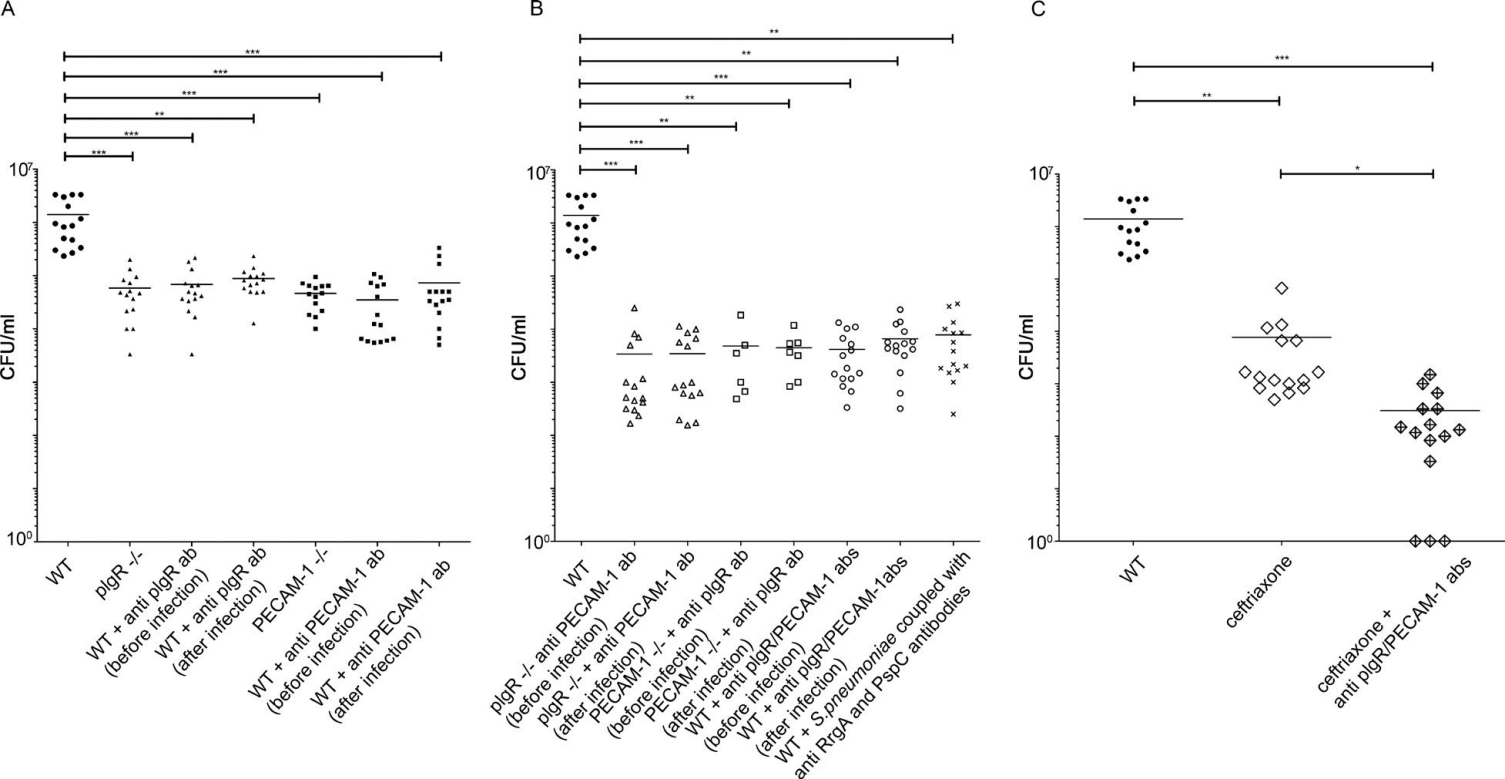
**Antibody treatment targeting PECAM-1 and/or pIgR reduces pneumococcal invasion of the brain in mice.** C57BL/6 mice were infected intravenously with the bioluminescent TIGR4 strain. Information on the pneumococcal strain and mouse strains used is stated in Material and methods, and information about antibodies is stated in Table S7. Each CFU value represents one mouse, and all values reflect the total population size of the experiment. Numbers of pneumococci (CFU/ml) found in the brain 14 h after infection are shown. Infected mice were compared with WT mice without any treatment (WT) in each graph. (A) WT mice (black circles) compared with mice in which only one adhesion receptor was absent or blocked with antibodies 1 h before or after infection, pIgR (black triangles) or PECAM-1 (black squares). (B) WT mice compared with mice in which both receptors were blocked simultaneously, using pIgR^−/−^ mice treated with anti–PECAM-1 antibody (empty triangles) or PECAM-1^−/−^ mice treated with anti-pIgR antibody (empty squares), or WT mice treated with both antibodies (anti-pIgR and anti–PECAM-1; empty circles). WT mice infected with *S. pneumoniae* coupled with anti-RrgA and anti-PspC antibodies (black crosses) are also shown. Antibodies were given 1 h before or after infection. (C) WT mice compared with mice treated with 100 mg/kg ceftriaxone (empty rhombus) administered intravenously 1 h after infection or mice treated with ceftriaxone combined with anti-pIgR and anti–PECAM-1 antibodies (crossed rhombus) 1 h after infection. In all panels, a nonparametric ANOVA test combined with the Dunn test (to make pairwise comparisons) was used. *, P < 0.05; **, P < 0.01; ***, P < 0.001. Horizontal lines indicate the mean.

Next, we studied the relevance of our findings in vivo using a murine bacteremia-derived meningitis model and a bioluminescent TIGR4 strain. We found that bacterial invasion of the brain was significantly reduced in pIgR^−/−^ and PECAM-1^−/−^ mice as compared with WT mice, indicating that both receptors are important for pneumococcal meningitis development (Fig. 5 A and Fig. S2 A). We then investigated whether we could prevent meningitis development using antibodies targeting pIgR or PECAM-1 in WT mice, and we found a significantly reduced pneumococcal invasion of the brain (∼10 times) as compared with nontreated mice (Fig. 5 A and Fig. S2 A). Importantly, reduced levels of pneumococci in the brain were observed irrespective of whether the antibodies were given to mice 1 h before or after pneumococcal challenge, suggesting that the antibodies could be used both as a treatment and prophylaxis. To study whether we could reduce the bacterial brain invasion even more by blocking both receptors, we used pIgR^−/−^ mice and PECAM-1 antibodies or PECAM^−/−^ mice with pIgR antibodies (Fig. 5 B and Fig. S2 B). We observed a pronounced reduction of the bacterial load (CFU) in the brain (>100-fold) as compared with WT mice (Fig. 5 B and Fig. S2 B). This reduction was independent on whether the antibodies were given before or after pneumococcal challenge. WT mice treated with both antibodies showed the same reduction of the bacterial load in the brain as was observed in mutant mice lacking pIgR or PECAM-1 and then treated with antibodies against the other receptor (Fig. 5 B and Fig. S2 B). Antibody treatment targeting both receptors was more effective in reducing pneumococcal entry into the brain than deleting the RrgA pilus adhesin (Iovino et al., 2016), likely because other bacterial adhesins such as PspC may recognize pIgR. No side effects in mice were observed after antibody treatment. As a control for the specificity of the antibodies, pIgR^−/−^ mice were treated with anti-pIgR antibody and then infected with pneumococci, and PECAM-1^−/−^ mice received anti–PECAM-1 antibody and then pneumococci. Similar numbers of pneumococci in the brain were observed as in pIgR^−/−^ and PECAM-1^−/−^ mice, respectively (Fig. S2, C and D). As an additional control, we also treated WT mice with EPCR antibody. As expected, no reduction of pneumococcal invasion of the brain was observed (Fig. S2 E).

In summary, these data show that antibody treatment targeting pIgR and PECAM-1 successfully prevent pneumococcal invasion of the brain. Furthermore, we studied whether antibodies to RrgA and PspC could reduce brain invasion. We found that when RrgA and PspC were coupled with the respective specific antibodies, they could no longer bind to pIgR or PECAM-1, resulting in a most significant reduction of pneumococcal invasion of the brain. As a negative control, we show that blockade of PspA on the bacterial surface did not affect the ability of pneumococci to invade the brain (Fig. S2 F).

Ceftriaxone is a third-generation cephalosporin that has been used previously in animal models for studies of meningitis (Congeni, 1984; Nau et al., 1999; Gerber et al., 2001; Kadurugamuwa et al., 2005). We infected WT mice with bioluminescent TIGR4 and treated them intravenously with ceftriaxone 1 h after infection. CFU levels in the brain were, as expected, significantly lower in antibiotic-treated mice than in WT mice (Fig. 5 C). However, despite administration of antibiotic at an early stage of infection, some pneumococci survived and invaded the brain (∼7 × 10^3^ CFU/ml; Fig. 5 C). To study whether antibody treatment could further reduce bacterial invasion from the bloodstream to the brain during treatment with antibiotics, we administered anti–PECAM-1 and pIgR antibodies in combination with ceftriaxone 1 h after infection. Indeed, pneumococcal invasion into the brain was further reduced and, in some mice, completely abolished (Fig. 5 C and Fig. S2 G), suggesting that bacteria surviving antibiotic treatment did not invade the brain when PECAM-1 and pIgR were blocked using antibody treatment.

In conclusion, we show that pneumococci colocalize with the endothelial receptors pIgR and PECAM-1 in human brain biopsies, and we identify the pilus protein RrgA as the major interacting partner with both receptors, whereas PspC interacts only with pIgR, but to a lesser extent than RrgA. We show that treatment with blocking antibodies targeting the two receptors, before or after pneumococcal challenge, protects mice from pneumococcal entry into the brain. Our data suggest that antibodies against pIgR and PECAM-1 can be used to prevent the development of pneumococcal meningitis.

## Material and methods

### Pneumococcal strains and growth conditions

The bioluminescent TIGR4 strain of serotype 4 (Xenogen 3; PerkinElmer) was used in the in vivo mouse experiments. For in vitro experiments, nonbioluminescent WT TIGR4 and isogenic mutants in RrgA (TIGR4Δ*rrgA*; Nelson et al., 2007) or PspC (TIGR4Δ*pspC*; Dieudonné-Vatran et al., 2009) were used. Additionally, six clinical pneumococcal isolates were studied collected from patients who died of pneumococcal meningitis. All pneumococci were grown in Todd–Hewitt broth with 0.5% yeast extract (THY) at 37°C and growth was monitored by measuring the optical density (OD) at 600 nm with a spectrophotometer. At OD_600_ =0.25–0.30 bacteria were harvested and collected in 1ml aliquots. For mouse experiments, bioluminescent TIGR4 aliquots were centrifuged at 10,000 rpm for 3 min and the pellet resuspended in 1 ml of sterile PBS. Serial dilutions were made in sterile PBS and plated on blood-agar plates to determine the dilutions required for 5 × 10^7^ CFUs for intravenous challenge.

### Human cell lines

HBMECs were obtained from J.M. van Dijl (University Medical Center Groningen, Groningen, Netherlands) and cultivated as previously described (Iovino et al., 2013).

### Mouse experiments

All animal experiments were approved by the local ethical committee (Stockholms Norra djurförsöksetiska nämnd). The bacteremia-derived meningitis model was performed as previously described (Iovino et al., 2013, 2016). All mice used (WT C57BL/6 and knockout mice [pIgR^−/−^ and PECAM-1^−/−^]) were 6–7 wk old. pIgR^−/−^ and PECAM-1^−/−^ mice were both in the C57BL/6 background. pIgR^−/−^ breeding pairs were obtained from the Institute of Microbiology, ETH Zurich, Switzerland, and PECAM-1^−/−^ breeding pairs were obtained from the Blood Research Institute, Milwaukee, WI. For intravenous challenge, 200 µl of 5 × 10^7^ CFU pneumococci was injected intravenously into the tail vein, and mice were sacrificed at 14 h after bacterial challenge. After sacrifice, unattached bacteria in the bloodstream were removed by perfusion with sterile PBS in the right ventricle via the vena cava until complete blood removal. After perfusion, mice were imaged using the IVIS Spectrum Imaging System and bioluminescent signal from the brain of the mice was imaged (for quantification of the bioluminescent signal, see Quantification of bioluminescent and fluorescent signal). After harvesting, one half of the brain (from five mice/group) was cryopreserved and stored with Shandon Cryomatrix (Thermo Fisher Scientific) at −80°C, whereas the other half, together with the full brains of the other mice, was used to prepare homogenate samples (see Preparation of mouse tissue homogenates).

For antibody treatment in mice, 20 µg/ml receptor-specific antibodies (listed in the Antibodies and isotype controls section) was administered intravenously in a volume of 200 µl. All antibodies were diluted in sterile PBS. Antibodies were administered either 1 h before or after challenge with pneumococci. Anti-RrgA, PspC, and PspA antibodies (Table S5) were used to couple the bacteria (see description in Immunofluorescent stainings) 1 h before challenge of the mice. 100 mg/kg ceftriaxone (Sigma-Aldrich) was administered intravenously (either alone or in combination with anti-pIgR and anti–PECAM-1 antibodies) 1 h after challenge with pneumococci.

### Preparation of mouse tissue homogenates and bacteria count in the brain

After harvesting, mouse brains were kept in 1 ml cold sterile PBS and homogenized using a cell strainer with a 100-µm filter (Falcon). Serial dilutions were made in sterile PBS and plated on blood-agar places for CFU counts.

### Antibodies and isotype controls

All antibodies used for immunofluorescent detection, Western blot, and in vivo experiments are listed in Tables S1, S2, S3, S4, S5, S6, and S7. Immunofluorescent detection was performed using antibody combinations diluted in sterile PBS with 5% FCS (Biochrom) as follows. As isotype controls, rabbit and mouse IgG (Innovative Research), goat IgG (Santa Cruz Biotechnology, Inc.) and rat IgGs (Sigma-Aldrich) were used at the same dilution as those for specific primary antibodies where no fluorescent signal was detected. Antibodies used for Western blot experiments were diluted in PBS-T supplemented with 1% dry milk.

### Immunofluorescent stainings

Tissue sections were fixed with acetone for 10 min and dried. Incubation with the antibodies was performed for 1 h, in the dark in case of fluorophore-labeled antibodies. Slides were washed twice in PBS for 5 min between the incubations. Once the staining was completed, Vectashield solution (Vector Laboratories) was added to each section before the coverslip was applied.

Bacterial staining of RrgA, PspC (bacterial proteins), and pIgR, PECAM-1 (HBMEC receptors) was performed as previously described (Iovino et al., 2016). In brief, after growth in THY medium, pneumococci were incubated with HBMEC lysate for 1 h. After incubation, the bacteria were washed three times with PBS-T, and immunofluorescent staining for RrgA/PspC and PECAM-1/pIgR was performed on bacterial pellets.

### High-resolution microscopy imaging and 3D model reconstruction

Microscopic analysis was performed using a DV Elite microscope (Applied Precision Ltd.). The z-stack images were acquired using a scientific complementary metal-oxide semiconductor (sCMOS) camera and processed with SoftWoRx imaging program (Applied Precision Ltd.). Images (z-stacks) taken with the DV Elite Imaging System have been rotated using the 3D Volume Viewer function of the imaging software SoftWoRx.

### STED super-resolution imaging

STED imaging was performed with an instrument from Abberior Instruments, built on a stand from Olympus (IX83), with a four-mirror beam scanner (Quad scanner; Abberior Instruments), and modified for two-color STED imaging. Two fiber-coupled, pulsed (20-MHz) diode lasers emitting at 637 nm (LDH-D-C; PicoQuant AG) and 594 nm (Abberior Instruments) are used for excitation (alternating pixel mode, with the excitation by the two lasers and gating of detector alternating in each pixel during scanning to minimize cross-talk). The beam of a pulsed fiber laser (model PFL-P-30-775-B1R, 775 nm emission, 40 MHz repetition rate, 1.2-ns pulse width, 1.2-W maximum mean power, 30-nJ pulse energy; MPB) is reshaped by a phase plate (VPP-1c; RPC Photonics) into a donut profile and then used for stimulated emission. The three laser beams are overlapped and then focused by an oil-immersion objective (UPLSAPO 100XO, NA 1.4; Olympus) into the sample. The fluorescence is collected through the same objective, separated from the excitation path via a dichroic mirror, passed through a motorized confocal pinhole (MPH16, set at 50-µm diameter; Thorlabs) in the image plane, split by a dichroic mirror, and then detected by two single-photon counting detectors (SPCM-AQRH-13; Excelitas Technologies), equipped with separate emission filters (FF01-615/20 and FF02-685/40-25; Semrock) and a common IR-filter (FF01-775/SP-25; Semrock) to suppress any scattered light from the STED laser. In this study, a spatial resolution (FWHM) of ∼25 nm could be reached. Image acquisition, including laser timing/triggering and detector gating, is controlled via a FPGA card and by the Imspector software (Abberior Instruments).

### Western blot analysis and quantification of protein expression

1 ml bacterial cultures (see Pneumococcal strains and growth conditions) was centrifuged at 10,000 rpm for 3 min, and the pellet was resuspended in 1× SDS-sample buffer (Thermo Fisher Scientific and Invitrogen) and boiled at 95°C for 5 min. Quality of each tissue homogenate was assessed by SDS-PAGE after Coomassie staining. Bacterial lysates were loaded onto a 10% NuPage Novex Bis-Tris Gel (Thermo Fisher Scientific and Invitrogen), and electroblotting was performed using the Bio-Rad Laboratories Trans-Blot Turbo Transfer System. Density of protein bands on Western blot membranes were measured with ImageJ (Schneider et al., 2012). Rectangles were drawn around each protein band, the intensity of pixels from the top of the rectangle to the bottom of the rectangle was generated, and the areas of each peak of pixel intensity were calculated. Protein signal values (pIgR and PECAM-1) were corrected for the pneumolysin loading control.

### PCR method

To assess the presence of *rrgA* and *pspC* genes in the clinical meningitis isolates, colony PCR was performed. Synthetic oligonucleotide primers (Sigma-Aldrich) used in PCR amplifications are listed below in List of primers used. PCR was performed using Fusion Flash Master Mix 2X (Thermo Fisher Scientific).

### Preparation of human brain sections

The formaldehyde-fixed paraffin-embedded brain tissue blocks were cut at 4-µm thickness and mounted on the slide glass (Starfrost). Ethical approval was obtained from the Academic Medical Center of Amsterdam, Netherlands.

### In vitro interaction studies between *S. pneumoniae* and endothelial receptors using HBMECs

Lysate of HBMECs was prepared as previously described (Iovino et al., 2014b). In brief, 250 µl RIPA lysis buffer was added to confluent HBMECs grown in T25 flasks (Starstedt). Cells were scraped and harvested and, after centrifugation at 13,000 rpm for 10 min at 4°C, the cell lysate in the supernatant was harvested. Quality of each tissue homogenate was assessed by SDS-PAGE after Coomassie staining. 100 µl HBMECs was added to 100 µl of the pneumococcal suspension in PBS (10^7^ CFU/ml), and the mixture was incubated at 4°C with gentle agitation for 1 h. After cold centrifugation, the supernatant was removed and the bacterial pellet was resuspended in LDS sample buffer 1X LDS-sample buffer (Thermo Fisher Scientific and Invitrogen) and boiled at 95°C for 5 min. Lysates were loaded onto a 10% NuPage Novex Bis-Tris Gel (Thermo Fisher Scientific and Invitrogen), and electroblotting was performed using the Trans-Blot Turbo Transfer System.

### Expression and purification of RrgA and PspA

Truncated *rrgA* encoding amino acids 39–868 was amplified by PCR from *S. pneumoniae* TIGR4 genomic DNA and ligated into pACYCDuet-1 vector (Novagen) to generate N-terminally 6x-His tagged RrgA_39–868_. Expression and purification was performed as described previously (Moschioni et al., 2010), with a few changes. In brief, T7 express competent *Escherichia coli* cells (New England Biolabs, Inc.) expressing 6xHis- RrgA_39–868_ were grown overnight at 16°C after induction of protein expression with isopropyl-β-d-thiogalactopyranoside at 1 mM final concentration. Cells were lysed by French press, and RrgA_39–868_ was purified by nickel affinity chromatography on His-Trap HP columns (GE Healthcare) according to the manufacturer's instructions. Fractions containing purified protein were pooled and dialyzed overnight against 20 mM Tris-HCl, pH 7.5, for subsequent ion-exchange chromatography essentially as described elsewhere (Moschioni et al., 2010). Pooled fractions containing RrgA_39–868_ were dialyzed overnight against PBS and used for immunoprecipitation experiments.

Part of the *pspA* open reading frame was PCR amplified from genomic DNA from the *S. pneumoniae* TIGR4 strain using primers *pspA*-frw and *pspA*-rev. The amplicon included codon 32–774 plus the TAA stop codon, thus excluded the 31st first codons encoding the N-terminal signal peptide. The primers also included nonannealing overhang sequences complementary to a sequence in the pET21d vector. The pET21d vector (EMD Millipore) was PCR amplified with the pET21d-frw and pET21d-rev primers. The *pspA* amplicon and the pET21d amplicon were purified with QIAquick PCR Purification kit (QIAGEN) and mixed and incubated with DpnI restriction enzyme for 2 h at 37°C to degrade the backbone template vector. An aliquot from the mixture was used to transform XL gold cells (Agilent Technologies), and the insert was integrated into the vector by in vivo homologous recombination (Bubeck et al., 1993; Mellroth et al., 2012). The plasmid was recovered from colonies isolated from ampicillin-containing Luria agar plates. The sequence of the *pspA*-pET21d expression vector was confirmed by DNA sequencing. For protein expression, competent Rosetta 2 cells (EMD Millipore) were transformed with the *pspA*-pET21d, and protein expression and purification followed the same procedure previously described for purification of LytA (Mellroth et al., 2012).

### List of primers used

The following primers were used: *pspC* forward, 5′-CTTCTTCATATGACAGAGAACGAGGGAGCTACCCAAGTA-3′; *pspC* reverse, 5′-CTTCTTCTCGAGCGCCATTGAACCATCAGTATTGTA-3′; *rrgA* forward, 5′-CGCGGATCCGAAAAAAGTAAGAAAGATATTTCAGAAGGCAGTTG-3′; *rrgA* reverse, 5′-CGCGTCGACTTACGGATGTTTCCGTGTGTATAATAGAACTC-3′; *aroE* forward, 5′-AAGCTTGATGGCTATACACG-3′; *aroE* reverse, 5′-ATCCATGCCCACACTGG-3′; *pspA* forward, 5′-TATACCTTGGCTAGCAGATGGAAGAATCTCCACAAGTTGT-3′; *pspA* reverse, 5′-AGCAGCCGGATCCTCGAGTTAAACCCATTCACCATTGG-3′; pET21 forward, 5′-CTCGAGGATCCGGCTGCTAAC-3′; and pET21 reverse, 5′-CATCTGCTAGCCAAGGTATA-3′.

### Coimmunoprecipitation experiments between pneumococcal proteins and host receptors

For each coimmunoprecipitation experiment, 50 µl magnetic Dynabeads (Thermo Fisher Scientific and Invitrogen) was transferred into an Eppendorf tube. The tube was placed on a magnet to separate beads from the solution, the supernatant was removed, and the tube displaced from the magnet. 100 µl anti-RrgA antiserum (1–5 µg) diluted in PBS was added to the beads and incubated rotating for 30 min at room temperature. The tube was placed on the magnet, the supernatant was removed, the bead–antibody complex was washed with PBS-T, the tube was placed on the magnet to remove the supernatant, and 100 µl antigen (1–5 µg) was added. The bead–antibody–antigen complex was incubated with rotation for 30 min at room temperature. The tube was placed on the magnet to remove the supernatant, and bead–antibody–antigen was washed with PBS-T. The tube was placed on the magnet to remove the supernatant, and either mouse pIgR (catalog no. CF-2800; R&D Systems) or PECAM-1 (catalog no. 3628-PC; R&D Systems) was added, and the bead–antibody–antigen–protein complex was incubated with rotation for 30 min at room temperature. The tube was placed on magnet to remove the supernatant, and the complex was washed with PBS-T. LDS sample buffer 1X (Thermo Fisher Scientific and Invitrogen) was added, and the complex was boiled at 95°C for 10 min. Beads were finally separated from proteins using the magnet, and protein detection was performed using SDS-PAGE and Western blot (see Western blot analysis). As a negative control, anti–GST antibody (Sigma-Aldrich) was coupled with beads and the same coimmunoprecipitation experiment was performed with pIgR and PECAM-1.

### Quantification of bioluminescent and fluorescent signal

Bioluminescence signal (data shown in Fig. S2) was quantified using ImageJ (Schneider et al., 2012). Bioluminescent signal was selected using the function Image-Adjust-Color Threshold. RGB Profile Plot was generated for each image taken with the IVIS Spectrum System, and intensities of blue/green/red colors were measured separately. Intensity of RGB (red, green, blue) colors was plotted in histograms displaying on the y axis the total RGB color intensity, and each column was divided into three parts (three colors: red, green, and blue) according to the intensity of each color. The color scale with the distinction of red (very severe infection), green (severe infection), and blue (mild infection) color range is shown. Fluorescent signal (data shown in Fig. S2, A–G) was quantified by ImageJ (Schneider et al., 2012). Fluorescent signal for each separate fluorescent channel was selected using the function Image-Adjust-Color Threshold, and area covered by each fluorescent signal was measured using the function Analyze-Measure.

### Colocalization analysis

Using the images obtained with Delta Vision Elite Imaging System, colocalization between two separate fluorescent signals was performed using ImageJ (National Institutes of Health; Schneider et al., 2012) Colocalization plug-in function turns all colocalized pixels in white color. Quantification of the colocalized pixels was performed using the function Image-Adjust-Color Threshold to select the area of colocalization and the area was measured using the function Analyze-Measure.

### Statistical analysis

For multiple comparisons, the nonparametric ANOVA test was used to assess the presence of the differences between the groups, and then the Dunn test was used to make pairwise comparisons.

### Supplemental material

Fig. S1 includes additional data from the high-resolution immunofluorescent analysis of human brain biopsies. Fig. S2 shows IVIS imaging analysis detecting bioluminescent pneumococci in the brain in vivo mice. Tables S1, S2, S3, S4, S5, S6, and S7 include primary and secondary antibodies, markers, and fluorophores for in vitro and mouse-specific antibodies for in vivo experiments.

## Supplementary Material

Supplemental Materials (PDF)
