# Supplementary material for: pIgR and PECAM-1 bind to pneumococcal adhesins RrgA and PspC mediating bacterial brain invasion
Source: J Exp Med. 2017 Jun 5;214(6):1619–30. doi: 10.1084/jem.20161668 (PMC5461002; doi:10.1084/jem.20161668)
Supplement: Supplemental Materials (PDF) [file JEM_20161668_sm.pdf]

SUPPLEMENTAL MATERIAL

Iovino et al., https://doi.org/10.1084/jem.20161668

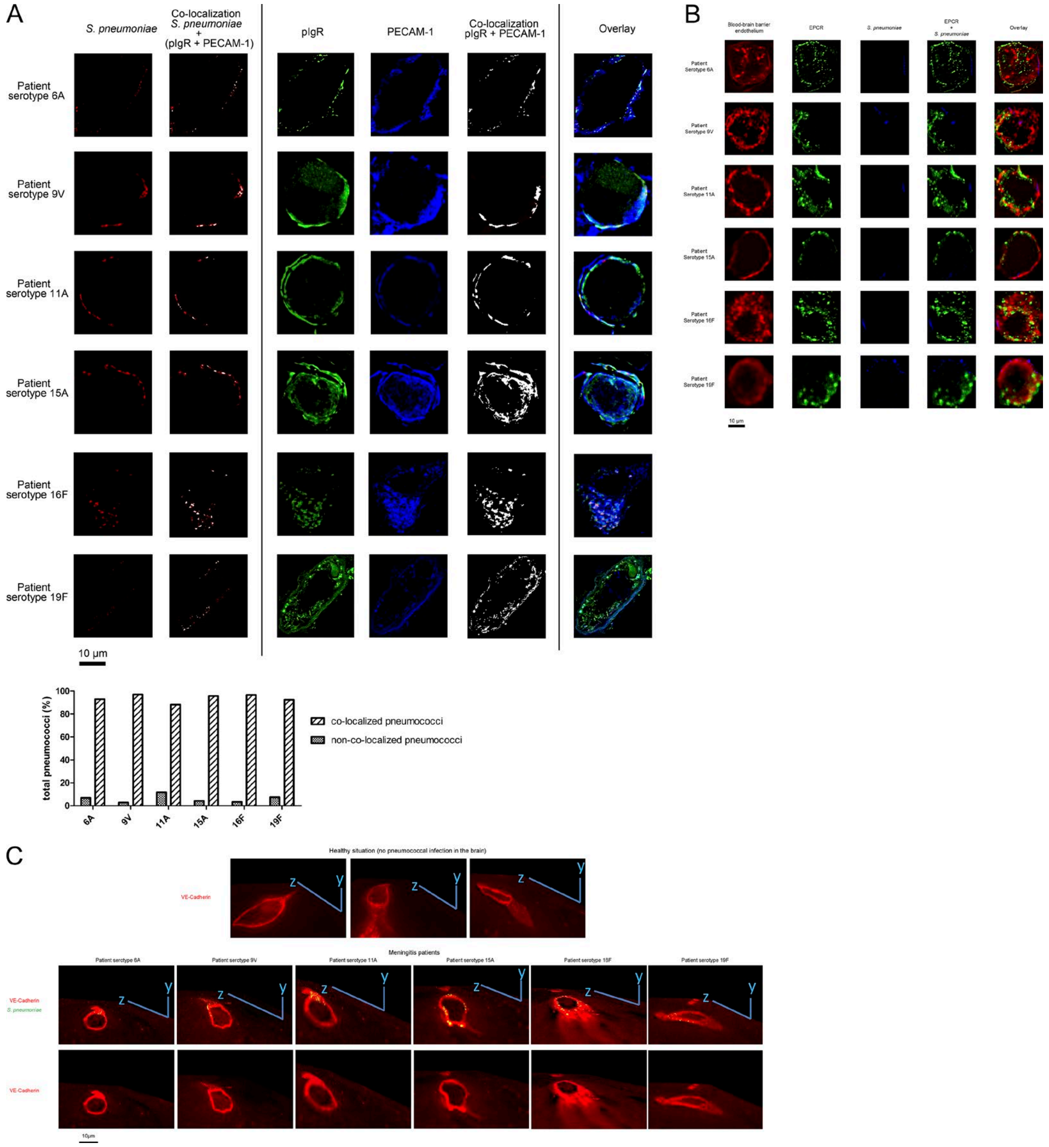

Figure S1. **Additional data from high-resolution immunofluorescent microscopy imaging of brain biopsy analysis.** (A) Biopsies from meningitis patients showing that pIgR and PECAM-1 are expressed on the BBB endothelium and colocalize with pneumococci. Immunofluorescent stainings, using the high-resolution Delta Vision Elite Imaging System, of six human biopsies from patients who died of pneumococcal meningitis caused by different serotypes. Each row shows images related to the section of one blood vessel selected as a representative among 50 blood vessels/patient imaged. (Left) Immunofluorescent detection of *S. pneumoniae* (red) and bacteria that colocalize with pIgR and PECAM-1 (white). (Middle) Immunofluorescent staining of pIgR (green), PECAM-1 (blue), and colocalization of pIgR and PECAM-1 (white). (Right) Immunofluorescent staining of PECAM-1 (blue), pIgR (green), and *S. pneumoniae* (red). The overlay shows that most pneumococci colocalize with both PECAM-1 and pIgR simultaneously (white; as shown in the middle panel) or with PEC AM-1 or pIgR only. (Graph) Calculation of the percentage of pneumococci that colocalize with pIgR, PECAM-1, or both receptors among all pneumococci that adhered to the BBB (set to 100%). For each serotype, the total percentages of colocalized and noncolocalized bacteria were calculated using the total amounts of colocalized and noncolocalized bacteria in 50 blood vessels/patient imaged. (B) Pneumococci do not colocalize with EPCR on the BBB endothelium in human brain biopsies. High-resolution immunofluorescent microscopy was performed on brain tissue sections from six meningitis patients. Each image (one for each patient) shows the section of a blood vessel selected as representative among 50 blood vessels per patient studied. Immunofluorescent detection of BBB endothelium (red), EPCR (green), and *S. pneumoniae* (blue) show that pneumococci do not colocalize with EPCR. (C) The passage of pneumococci across the BBB occurs without major disruptions of the vascular endothelium. 3D model images (z-stacks) taken with the Delta Vision Elite Imaging System. The yz axes show the angle of rotation of each image. Each image shows one representative image per each patient, including the three images of the uninfected brains (each image is representative of each uninfected patient). The panel "VE-Cadherin *S. pneumoniae*" shows bacteria (green) that adhered to the vascular endothelium (red), the panel "VE-Cadherin" shows the same images only with the VE-cadherin fluorescent signal showing the continuous endothelial layer also in the vessels in which bacteria were detected, indicating no major disruptions of the endothelium caused by the bacterial infection. Each image (one for each patient) shows the section of a blood vessel selected as a representative among the 10 blood vessels/patient analyzed.

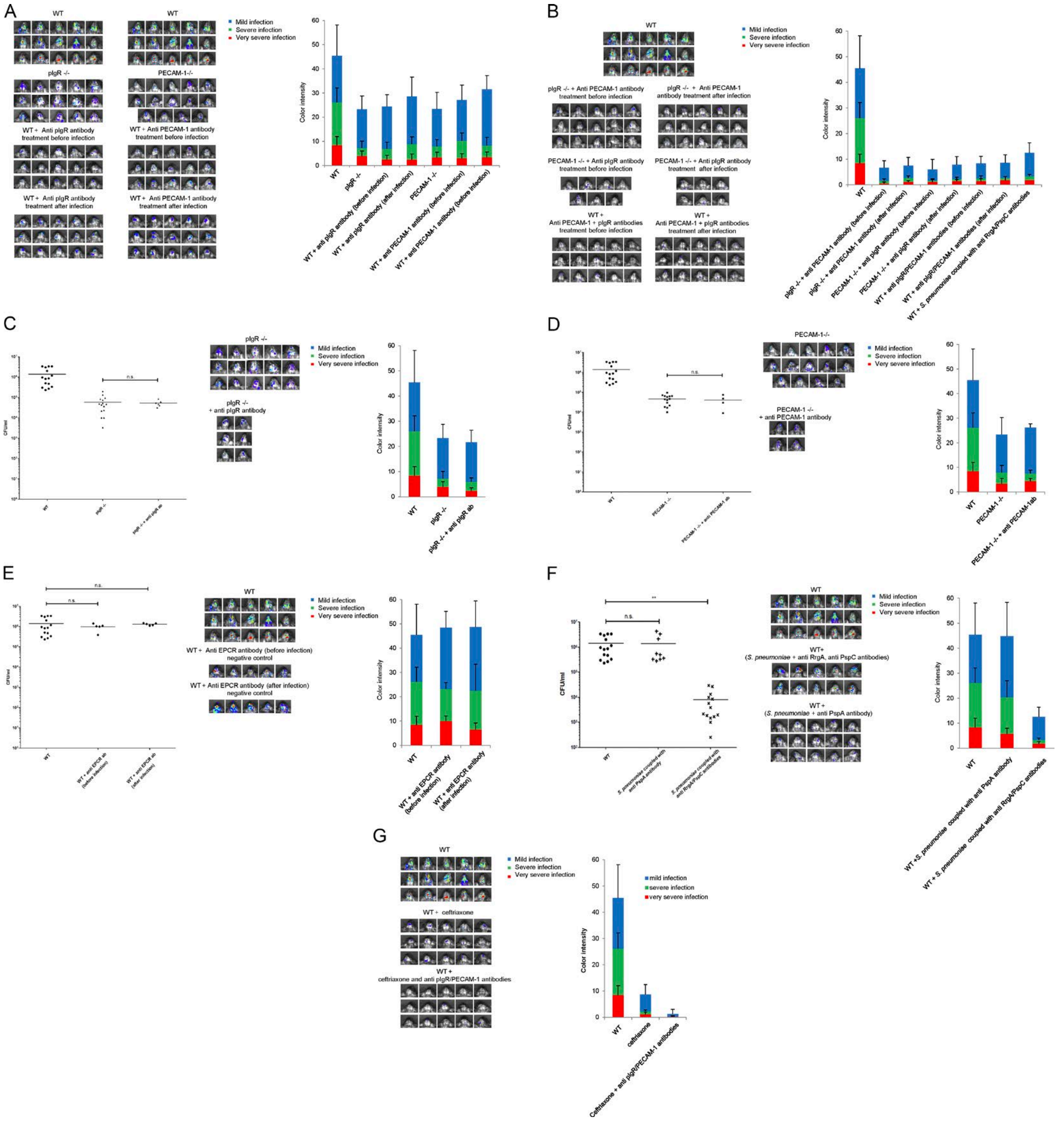

Figure S2. **IVIS imaging analysis detecting bioluminescent pneumococci in the brain in vivo.** (A) IVIS imaging analysis detecting bioluminescent pneumococci and quantification of the signal intensity in the brain of mice in the absence or blockade of one receptor (before and after infection). (B) IVIS imaging analysis detecting bioluminescent pneumococci and quantification of the signal intensity in the brain of mice in the absence or blockade of both receptors (before and after infection). (C) Numbers of pneumococci found in the brain 14 h after intravenous infection with bioluminescent TIGR4 of pIgR$^{-/-}$ mice treated with anti-pIgR antibody. The number of pneumococci detected was similar to those found in the brain of pIgR$^{-/-}$ mice; IVIS imaging analysis detecting bioluminescent pneumococci and quantification of the signal intensity in the brain of mice. (D) Numbers of pneumococci found in the brain 14 h after intravenous infection with bioluminescent TIGR4 of PECAM-1$^{-/-}$ mice treated with anti–PECAM-1 antibody. The number of pneumococci detected were similar to those found in the brain of PECAM-1$^{-/-}$ mice; IVIS imaging analysis detecting bioluminescent pneumococci and quantification of the signal intensity in the brain of mice. (E) Numbers of pneumococci found in the brain 14 h after intravenous infection with bioluminescent TIGR4 of WT mice treated with anti-EPCR antibody (before or after the infection). The number of pneumococci detected were similar to those found in the brain of non-treated WT mice; IVIS imaging analysis detecting bioluminescent pneumococci and quantification of the signal intensity in the brain of mice. (F) Number of pneumococci found in the brain 14 h after intravenous infection with bioluminescent TIGR4 coupled with anti-RrgA and anti-PspC antibodies or anti-PspA antibody compared with infection with uncoupled bioluminescent TIGR4; IVIS imaging analysis detecting bioluminescent pneumococci and quantification of the signal intensity in the brain of mice. **, $P < 0.01$. (G) Numbers of pneumococci found in the brain 14 h after intravenous infection with bioluminescent TIGR4 of WT mice infected with bioluminescent TIGR4 intravenously and then treated with ceftriaxone only or with ceftriaxone and antibodies to the receptors, pIgR and PECAM-1, 1 h after infection; IVIS imaging analysis detecting bioluminescent pneumococci and quantification of the signal intensity in the brain of mice. Error bars show standard deviations. In each graph, each column shows the mean value of the intensity of the bioluminescent signals in the brain of the mice, and each column has three means and three standard deviations (three error bars, as there are three colors that have been quantified: red, green, and blue). Each standard deviation was calculated using the intensity values of each color in the brain of each mouse used in the experiment. The horizontal bars in C–F show the mean values.

Table S1. **High-resolution microscopy (Delta Vision Elite Imaging System): Primary antibodies and markers**

Antibody	Antigen	Reactivity	Host	Dilution	Company	Catalog no.
DyLight 594–labeled LEL	Endothelium marker	Mouse, human	Lectin	1:200	Vector Laboratories	DL-1177
Anti-pIgR	pIgR	Human	Goat	1:50	R&D Systems	AF2717
Anti–PECAM-1	PECAM-1	Human	Mouse	1:50	Dako	M082329-2
Anti–*S. pneumoniae*	Capsule (serotype specific)	*S. pneumoniae* capsule	Rabbit	1:50	Statens Serum Institute	16323 (serotype 6A), 16937 (serotype 9V), 16944 (serotype 11A), 16951 (serotype 15A), 16954 (serotype 16F), 16962 (serotype 19F)
Anti-NG2	Pericytes	Mouse, human	Rabbit	1:50	Abcam	ab83178
Anti-PAFR	PAFR	Mouse, human	Rabbit	1:50	Abcam	ab104162
Anti-EPCR	EPCR	Human	Goat	1:50	R&D Systems	AF2245
Anti–VE-cadherin	VE-cadherin	Human	Mouse	1:50	Cell Signaling Technology	2500

Table S2. **High-resolution microscopy (Delta Vision Elite Imaging System): Secondary antibodies and fluorophores**

Antibody	Reactivity	Host	Detection	Dilution	Company	Catalog no.
Alexa Fluor 488 donkey anti–goat	Goat IgGs	Donkey	pIgR; EPCR	1:500	Thermo Fisher Scientific; Invitrogen	A-11055
Zenon mouse IgG labeling kit	Mouse IgGs	Not applicable	PECAM-1 (when anti-pIgR antibody was used in the same staining)	1:500	Thermo Fisher Scientific; Invitrogen	Z25000
Alexa Fluor goat anti–rat 488	Rat IgGs	Goat	PECAM-1 (when DyLight 594–labeled LEL was used in the same staining)	1:500	Thermo Fisher Scientific; Invitrogen	A-11006
Zenon rabbit IgG labeling kit	Rabbit IgGs	Not applicable	*S. pneumoniae*	1:500	Thermo Fisher Scientific; Invitrogen	Z25307
Alexa Fluor 488 goat anti–rabbit	Rabbit IgGs	Goat	PAFR (when DyLight 594–labeled LEL or anti *S. pneumoniae* antibody were used in the same staining); *S. pneumoniae* (when anti–VE-Cadherin antibody was used in the same staining)	1:500	Thermo Fisher Scientific; Invitrogen	A-11008
Alexa Fluor goat anti–mouse 594	Mouse IgGs	Goat	VE-Cadherin	1:500	Thermo Fisher Scientific; Invitrogen	A-11005

Labeling kits were used when two secondary antibodies would have cross-reacted in the same staining.

Table S3. **STED super-resolution microscopy: Primary antibodies and markers**

Antibody	Antigen	Reactivity	Host	Dilution	Company	Catalog no.
DyLight 594–labeled LEL	Endothelium marker	Mouse, human	Lectin	1:200	Vector Laboratories	DL-1177
Anti-pIgR	pIgR	Mouse, human	Rat	1:50	Abcam	ab170321
Anti–PECAM-1	PECAM-1	Mouse	Rat	1:50	BD	550274
Anti–*S. pneumoniae*	Capsule (serotype specific)	*S. pneumoniae* capsule	Rabbit	1:50	Statens Serum Institute	16323 (serotype 6A), 16937 (serotype 9V), 16944 (serotype 11A), 16951 (serotype 15A), 16954 (serotype 16F), 16962 (serotype 19F)
Anti-RrgA	RrgA	*S. pneumoniae*	Rabbit	1:50	Novartis	Not applicable
Anti-PspC	PspC	*S. pneumoniae*	Mouse	1:50	Novartis	Not applicable

Table S4. **STED super-resolution microscopy: Secondary antibodies**

Antibody	Reactivity	Host	Detection	Dilution	Company	Catalog no.
Alexa Fluor 594 goat anti–rat	Rat IgGs	Goat	pIgR; PECAM-1 (when anti–*S. pneumoniae* or anti-RrgA/PspC antibodies were used in the same staining)	1:500	Thermo Fisher Scientific; Invitrogen	ABIN965007; A-11007
ATTO 647N goat anti–rabbit	Rabbit IgGs	Goat	RrgA (when anti–PECAM-1 or anti-pIgR antibodies were used in the same staining); *S. pneumoniae* (when anti–PEC AM-1 or anti-pIgR antibodies were used in the same staining)	1:500	Sigma-Aldrich	40839
ATTO 647 goat anti–mouse	Mouse IgGs	Goat	PspC (when anti–PECAM-1 or anti-pIgR antibodies were used in the same staining)	1:500	Sigma-Aldrich	50185
ATTO 647 goat anti–rat	Rat IgGs	Goat	PECAM-1; pIgR (when DyLight 594–labeled LEL was used in the same staining)	1:500	Antibodies-online	ABIN965007

Table S5. **Western blot experiments: Primary antibodies**

Antibody	Antigen	Reactivity	Host	Dilution	Company	Catalog no.
Anti-pIgR	pIgR	Human	Goat	1:1,000	R&D Systems	AF2717
Anti-pIgR	pIgR	Mouse	Goat	1:1,000	R&D Systems	AF2800
Anti–PECAM-1	PECAM-1	Human	Mouse	1:1,000	Dako	M082329-2
Anti–PECAM-1	PECAM-1	Mouse	Rat	1:1,000	BD	550274
Anti-RrgA	RrgA	*S. pneumoniae*	Rabbit	1:1,000	Novartis	Not applicable
Anti-PspC	PspC	*S. pneumoniae*	Mouse	1:1,000	Novartis	Not applicable
Anti-pneumolysin	Pneumolysin	*S. pneumoniae*	Mouse	1:1,000	Abcam	ab71810
Anti-EPCR	EPCR	Human	Goat	1:1,000	R&D Systems	AF2245
Anti-PspA	PspA	*S. pneumoniae*	Goat	1:1,000	Santa Cruz Biotechnology, Inc.	sc-17483
Anti-LytA	LytA	*S. pneumoniae*	Rabbit	1:1,000	Agro-bio	Not applicable
Anti-GST	GST (to couple Dynabeads-coupled GST)	*S. pneumoniae*	Mouse	1:1,000	Sigma-Aldrich	ABN116
Anti-GST	GST (to detect GST in immunoprecipitation experiments)	*S. pneumoniae*	Rabbit	1:1,000	Abcam	ab19256

Table S6. **Western blot: Secondary antibodies**

Antibody	Reactivity	Host	Detection	Dilution	Company	Catalog no.
HRP-conjugated donkey anti–goat	Goat IgGs	Donkey	pIgR; PspA	1:5,000	Thermo Fisher Scientific; Invitrogen	A15999
HRP-conjugated goat anti–rat	Rat IgGs	goat	PECAM-1	1:5,000	GE Healthcare	NA935V
HRP-conjugated goat anti–rabbit	Rabbit IgGs	Goat	RrgA; LytA; GST	1:5,000	GE Healthcare	RPN4301
HRP-conjugated goat anti–mouse	Mouse IgGs	Goat	Pneumolysin; PspC; GST	1:5,000	GE Healthcare	NXA931

Table S7. **In vivo experiments: Mouse-specific antibodies**

Antibody	Antigen	Host	Concentration	Company	Catalog no.
Anti–PECAM-1	PECAM-1	Rat	20 µg/ml	BD	550274
Anti-pIgR	pIgR	Rat	20 µg/ml	Abcam	ab170321
Anti-EPCR	EPCR	Goat	20 µg/ml	R&D Systems	AF2245
Anti-PspA	PspA	Goat	1:50 (used to couple pneumococci before infection in the mice)	Santa Cruz Biotechnology, Inc.	sc-17483

When mice were infected with pneumococci that were previously coupled with both anti-RrgA and anti-PspC antibodies, the antibodies were used at the same dilution as shown in Table S3.
